# Habitat disturbance influences the skin microbiome of a rediscovered neotropical-montane frog

**DOI:** 10.1186/s12866-020-01979-1

**Published:** 2020-09-22

**Authors:** Randall R. Jiménez, Gilbert Alvarado, José Sandoval, Simone Sommer

**Affiliations:** 1 Institute of Evolutionary Ecology and Conservation Genomics, University of Ulm, Albert-Einstein Allee 11, 89069 Ulm, Germany; 2 Laboratory of Comparative Wildlife Pathology, School of Veterinary Medicine and Animal Sciences, University of São Paulo, Av. Orlando Marques de Paiva 87, São Paulo, Brazil; 3 Laboratory of Experimental and Comparative Pathology (LAPECOM), Biology School, University of Costa Rica, San José, Costa Rica

**Keywords:** Skin bacterial diversity, 16S rRNA amplicon sequencing, Land-use change, Anthropogenic disturbance, *Batrachochytrium dendrobatidis* (*Bd*), Amphibian health implications and conservation

## Abstract

**Background:**

The skin microbiome serves as a first line defense against pathogens in vertebrates. In amphibians, it has the potential to protect against the chytrid fungus *Batrachochytrium dendrobatis* (*Bd*), a likely agent of amphibian declines. Alteration of the microbiome associated with unfavorable environmental changes produced by anthropogenic activities may make the host more susceptible to pathogens. Some amphibian species that were thought to be “extinct” have been rediscovered years after population declines in the late 1980s probably due to evolved *Bd*-resistance and are now threatened by anthropogenic land-use changes. Understanding the effects of habitat disturbance on the host skin microbiome is relevant for understanding the health of these species, along with its susceptibility to pathogens such as *Bd*. Here, we investigate the influence of habitat alteration on the skin bacterial communities as well as specifically the putative *Bd*-inhibitory bacterial communities of the montane frog *Lithobates vibicarius*. This species, after years of not being observed, was rediscovered in small populations inhabiting undisturbed and disturbed landscapes, and with continuous presence of *Bd*.

**Results:**

We found that cutaneous bacterial communities of tadpoles and adults differed between undisturbed and disturbed habitats. The adults from disturbed habitats exhibited greater community dispersion than those from undisturbed habitats. We observed a higher richness of putative *Bd*-inhibitory bacterial strains in adults from disturbed habitats than in those from undisturbed habitats, as well as a greater number of these potential protective bacteria with a high relative abundance.

**Conclusions:**

Our findings support the microbial “Anna Karenina principle”, in which disturbance is hypothesized to cause greater microbial dispersion in communities, a so-called dysbiosis, which is a response of animal microbiomes to stress factors that decrease the ability of the host or its microbiome to regulate community composition. On the positive side, the high richness and relative abundance of putative *Bd*-inhibitory bacteria may indicate the development of a defense mechanism that enhances *Bd*-protection, attributed to a co-occurrence of more than 30-years of host and pathogen in these disturbed habitats. Our results provide important insight into the influence of human-modified landscapes on the skin microbiome and health implications of *Bd*-survivor species.

## Background

All vertebrates are hosts to numerous symbiotic microorganisms that reside on their skin [1, 2]. The host depends on its natural microbial communities for different vital functions and can therefore contribute to maintaining host health (e.g. protection against pathogen invasion and colonization) [3–5]. Disruption of microbial communities beyond the natural range (i.e., dysbiosis) associated with environmental stress, such as unfavorable environmental changes caused by anthropogenic activities, may lead to an increased incidence of certain diseases in their hosts [6, 7]. Severe disruption of microbial communities caused by changes in the pattern of microbiota abundance and/or loss of beneficial bacterial members in the community can affect the functionality of the microbiome and make the host more susceptible to pathogens [8, 9]. Thus, dysbiosis caused by adverse factors can disrupt the performance benefits of host-associated microbiota and may contribute to unwanted collateral damage to hosts [7]. This means that it can threaten the survival of individuals and the growth of populations due to a cascade of negative health effects [10–13]. In fact, several recent studies have documented how skin and gut microbiomes from different wildlife species can be sensitive to habitat fragmentation and contaminants attributed to human activities [14–18].

In amphibians, the environment is a strong determinant of the skin microbiome, but less so than the evolutionary history and development [19, 20]. Different environmental conditions, together with the available pool of potential bacterial symbionts in the environment, have been shown to influence the host microbiome, which may result in distinct host bacterial communities across locations [21, 22]. Large-scale differences attributed to anthropogenic disturbance in the host environment (e.g. conversion of natural land use to agriculture, and habitat fragmentation) may alter abiotic factors such as soil and water properties, which in turn impact on the diversity of environmental microorganisms, including those that may colonize hosts [22]. Furthermore, as human activities also affect the physiology, ecology, and behavior of wildlife [23], this can cause simultaneous changes in the dynamics of the amphibian skin microbiome. These types of adverse conditions could cause a dysbiosis in the host microbiome that could have a negative impact on amphibian health [24]. Analysis of the effects of land use change (e.g., habitat disturbance by human activities) is an approach to understanding how microbial communities associated with hosts respond to adverse environmental conditions (e.g., Amato et al. [11] and Hughey et al. [25]).

Some neotropical amphibians that were thought to be extinct have been rediscovered years after the amphibian populations declined in the late 1980s [26–28]. These species have been found in isolated, small populations that persist in the presence of the fungal chytrid pathogen *Batrachochytrium dendrobatidis* (*Bd*) [28]. This pathogen is a likely causative agent of the amphibian declines [29, 30]. It is suggested that host recoveries after declines are not caused by attenuation of *Bd* and may be due to evolutionary changes in host response [31]. These hosts and *Bd* appear to be in an enzootic state (i.e. a period in which the host and pathogen coexist) that allows amphibians to recover, but they remain vulnerable to this pathogen as *Bd* remains as pathogenic as it was during catastrophic declines [28, 31]. Previous studies have highlighted the relevance of the host skin microbiome to the defense mechanisms of the amphibian skin against *Bd* [32, 33]. Therefore, understanding the impact of habitat disturbance on the host skin microbiome is relevant for understanding host resistance and susceptibility to pathogens such as *Bd* [24]. The latter is of great interest for the conservation of amphibians threatened by *Bd* that survive after their presumed extinction and are under the constant influence of human activities.

Our study focused on the Green-eyed frog (*Lithobates vibicarius*), which is a tropical mountain amphibian. This species declined dramatically throughout its historical range in the late 1980s, to the point of being considered possibly extinct, and probably due to an epizootic phase of *Bd* [28]. Surprisingly, years after its disappearance, some populations of the species have been rediscovered in the Cordillera Central and Cordillera de Tilarán in Costa Rica [28, 34]. The species now appears to persist with *Bd* in an enzootic phase [28]. In our study area, the species survives in small populations inhabiting undisturbed and disturbed landscapes, and with continuous presence of *Bd* (Alvarado et al. unpublished observations). Individuals in disturbed areas are exposed to human activities such as livestock and agriculture, which could potentially threaten the health of the species. We previously found that these surviving populations have a low prevalence of *Bd-*infection and a dynamic skin microbiome with putative *Bd*-inhibitory bacteria in our study area (see Jiménez et al. [35])

In this study, we examined the potential effects of anthropogenic disturbance on skin bacterial communities and putative *Bd*-inhibitory bacteria in *L. vibicarius*. We predicted that the composition of the skin bacterial community would differ between individuals in different habitat types (i.e. undisturbed and disturbed habitats). We also expected that individuals from disturbed habitats would possess a more prominent dispersion of the bacterial community, suggesting a dysbiotic state, as these types of stressors can impact the natural range of the microbiome through different mechanisms [6, 9]. Since *L. vibicarius* now appears to be able to persist despite the presence of *Bd* in our study area (see Jiménez et al. [35]) and amphibians harbor diverse bacterial communities that may provide protection against *Bd* [36, 37], we expected individuals in both habitat types to possess bacterial strains with putative *Bd*-inhibitory activity. However, as *Bd* infections in other amphibian species have been detected more frequently in areas with human disturbances [38, 39], we expected individuals from undisturbed and disturbed habitats to possess different distribution patterns of bacterial strains with putative *Bd*-inhibitory activity. In addition, we predicted a higher prevalence and relative abundance of putative *Bd*-inhibitory bacteria in individuals from disturbed habitats where *Bd* is suggested to be more present in amphibians and could be a developed potential mechanism to fight this pathogen [40, 41].

## Results

The skin microbiome of frog samples from undisturbed and disturbed habitats were used for investigating the influence of habitat disturbance on the skin bacterial communities in tadpoles and adults of *L. vibicarius*. We prepared two microbial 16S rRNA datasets according to the analysis, i.e., 1) tadpole dataset and 2) adult dataset (Additional file 1: Table S1). The number of sequences per sample in the “tadpole dataset” ranged from 10,845 to 56,159 and in the “adult dataset” from 9378 to 54,704. The tadpole dataset contained samples collected from five sites (undisturbed sites = 2, disturbed sites = 3) in 2016 and 2017, while adult dataset contained samples collected at six study sites (undisturbed sites = 3, disturbed sites = 3) in 2016 and 2017 (Additional file 1: Table S1).

### Skin bacterial alpha and beta diversity in tadpoles and adults of *Lithobates vibicarius* between undisturbed and disturbed habitats

Habitat type explained significant variation in the number of observed ASVs (χ^2^ = 6.54, *p* = 0.01) and the Shannon diversity (χ^2^ = 6.83, *p* = 0.008) in tadpoles (Additional file 2: Table S2). Both measures of alpha diversity were higher in undisturbed habitats than in disturbed habitats (number of observed ASVs: odds ratio (OR) = 1.36, 95% confidence intervals (95% CI) not overlapping 1, Shannon diversity: OR = 1.16, 95% CI not overlapping 1, Additional file 2: Fig. S1A-B). The phylogenetic diversity of the tadpoles was similar between undisturbed and disturbed habitats (χ^2^ = 1.08, *p* = 0.29, Additional file 2: Table S2, Additional file 2: Fig. S1C). We observed that year of survey influenced the number of observed ASVs and phylogenetic diversity in tadpoles (Additional file 2: Table S2).

We found no differences in alpha diversity of adults between undisturbed and disturbed habitats (number of observed ASVs: χ^2^ = 0.07, *p* = 0.79, Shannon diversity: χ^2^ = 1.61, *p* = 0.20, phylogenetic diversity, χ^2^ = 0.51, *p* = 0.47, Additional file 2: Table S2, Additional file 2: Fig. S1D-F). The three alpha diversity metrics in adults were not influenced by year of the survey (Additional file 2: Table S2).

**Fig. 1:**
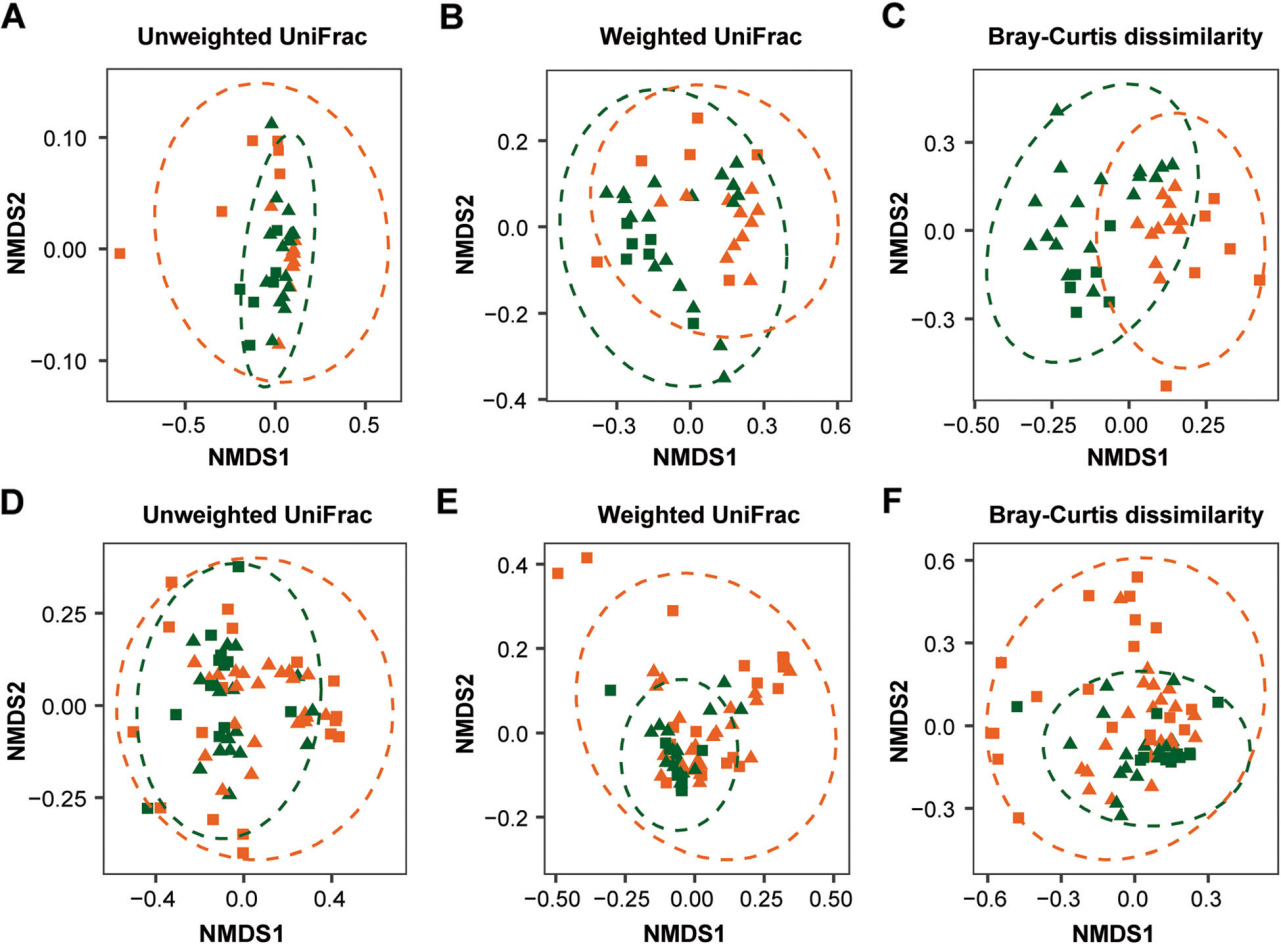
Beta diversity of *L. vibicarius* skin microbiota between undisturbed/disturbed habitats. The Non-Metric Multidimensional Scaling plots (NMDS) of the beta diversity of the microbiota of tadpoles (**a**-**c**) and adults (**d**-**f**) were based on unweighted UniFrac, weighted UniFrac and Bray-Curtis distance matrices. Each light point represents the bacterial community of an individual; point color indicates habitat types (green - undisturbed habitats and orange - disturbed habitats) and shape indicates year of survey (square - year 2016 and triangle - year 2017)

Regarding beta diversity, we observed differences in the ASV presence/absence and ASV abundance pattern when accounting phylogenetic lineages (unweighted UniFrac: R^2^ = 0.07, *p* = 0.001, weighted UniFrac: R^2^ = 0.14, *p* = 0.001, Additional file 2: Table S3, Fig. 1a-b) in tadpoles between undisturbed and disturbed habitats. In addition, non-phylogenetic abundance-based pattern was distinct in tadpoles between the two habitat types (Bray-Curtis dissimilarity: R^2^ = 0.14, *p* = 0.001, Additional file 2: Table S3, Fig. 1c). The year of the survey explained some variation for the three beta diversity metrics (Additional file 2: Table S3). Additionally, we observed that study sites explained part of the differentiation on ASVs abundance pattern of tadpoles (Bray-Curtis dissimilarity: R^2^ = 0.36, *p* = 0.001, Additional file 2: Table S4, Additional file 2: Fig. S2A). In adults, the skin bacteria presence/absence and abundance pattern when accounting for phylogenetic lineages differed between undisturbed and disturbed habitats (unweighted UniFrac: R^2^ = 0.08, *p* = 0.004, weighted UniFrac: R^2^ = 0.12, *p* = 0.049, Additional file 2: Table S3, Fig. 1d-e). Further, we observed a difference based on abundance pattern between habitat types when phylogenetic lineages were not considered (Bray-Curtis dissimilarity: R^2^ = 0.07, *p* = 0.001, Additional file 2: Table S3, Fig. 1f). The year of the survey explained part of the variation for the three beta diversity metrics (Additional file 2: Table S3). The NMDS plot of ASVs abundance pattern showed that adults clustered by study sites, and adults in sites without habitat disturbance clustered more tightly than those in sites with habitat disturbance (Additional file 2: Fig. S2B).

**Fig. 2:**
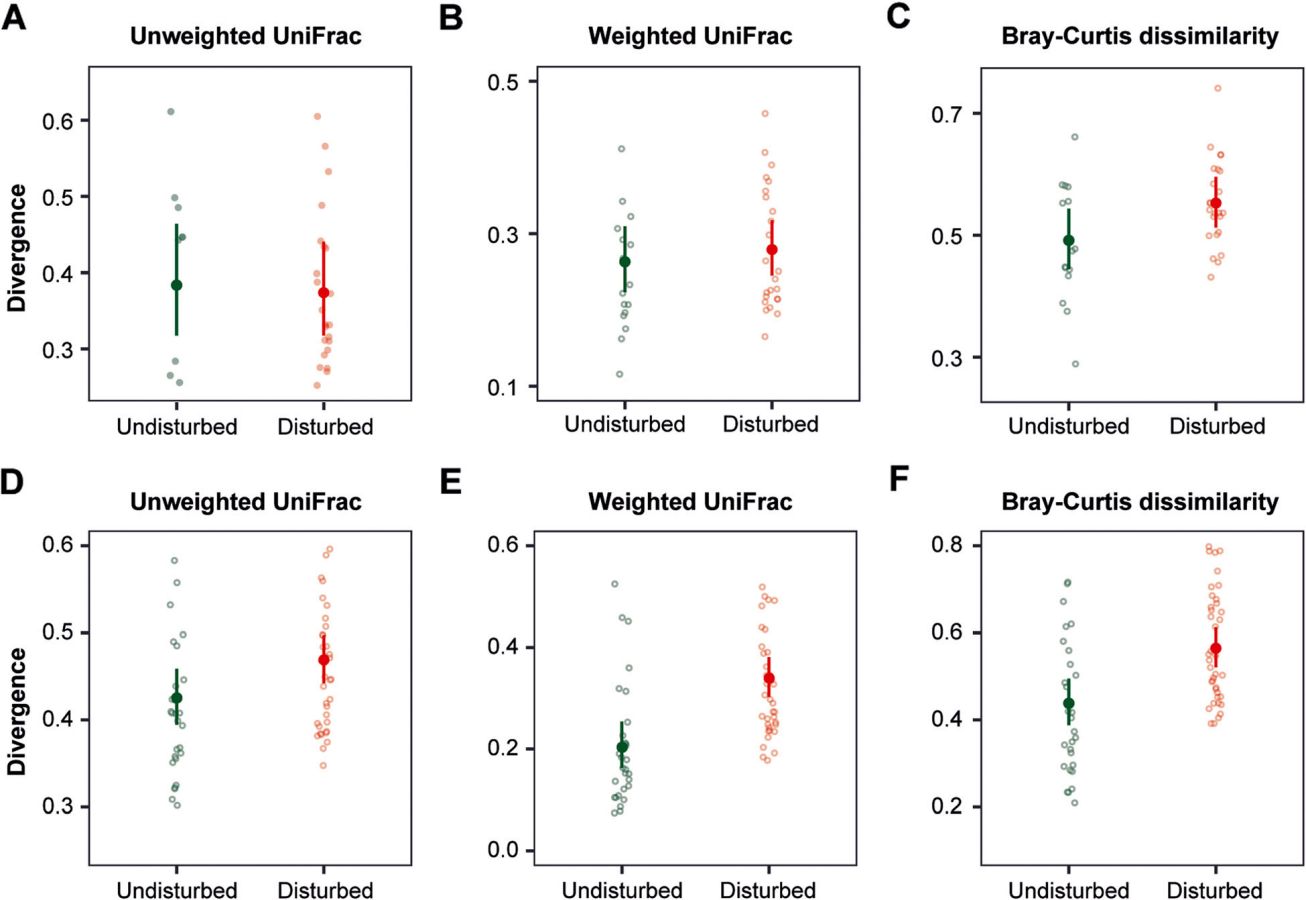
Beta diversity dispersion of *L. vibicarius* skin microbiota between undisturbed/disturbed habitats. Inter-individual distances from tadpoles (**a**–**c**) and adults (**d**–**f**) was calculated based on unweighted UniFrac, weighted UniFrac and Bray-Curtis distance matrices. Each light point represents the bacterial community of an individual; point color indicates habitat types (green - undisturbed habitats and orange - disturbed habitats)

Tadpoles across both habitat types did not show significantly different dispersion of the skin bacterial community, according to the unweighted UniFrac distance measure (χ^2^ = 0.04, *p* = 0.84, Fig. 2a) and weighted UniFrac distance measure (χ^2^ = 0.31, *p* = 0.57, Fig. 2b), as opposed to the Bray-Curtis dissimilarity, which showed a marginally different dispersion (χ^2^ = 3.41, *p* = 0.05, Fig. 2c). The skin microbiome of tadpoles from the disturbed habitats showed a more dispersed community among replicates than those from the undisturbed habitats (Bray-Curtis dissimilarity: OR = 1.12, 95% CI = 1.00–1.27). Adults from undisturbed and disturbed habitats showed a clear difference in the community dispersion of bacterial skin consortia, based on the unweighted UniFrac distance measure (χ^2^ = 4.08, *p* = 0.04), the weighted UniFrac distance measure (χ^2^ = 19.94, *p* < 0.0001) and the Bray-Curtis dissimilarity (χ^2^ = 12.49, *p* = 0.001). Specifically, adults from disturbed habitats showed a greater community dispersion than those from undisturbed habitats (Fig. 2d-f). We found a stronger difference of community dispersion between habitat types with the weighted UniFrac distance measure (OR = 1.67, 95% CI = 1.30–2.14) and Bray-Curtis dissimilarity (OR = 1.29, 95% CI = 1.11–1.49) than with the unweighted UniFrac distance measure (OR = 1.10, 95% CI = 1.00–1.21). The latter suggests a stronger effect of habitat disturbance on community dispersion for the ASV abundance pattern than for the ASV presence/absence.

### Differential abundance of bacterial ASVs in the skin of tadpoles and adults of *Lithobates vibicarius* across habitat types

**Fig. 3:**
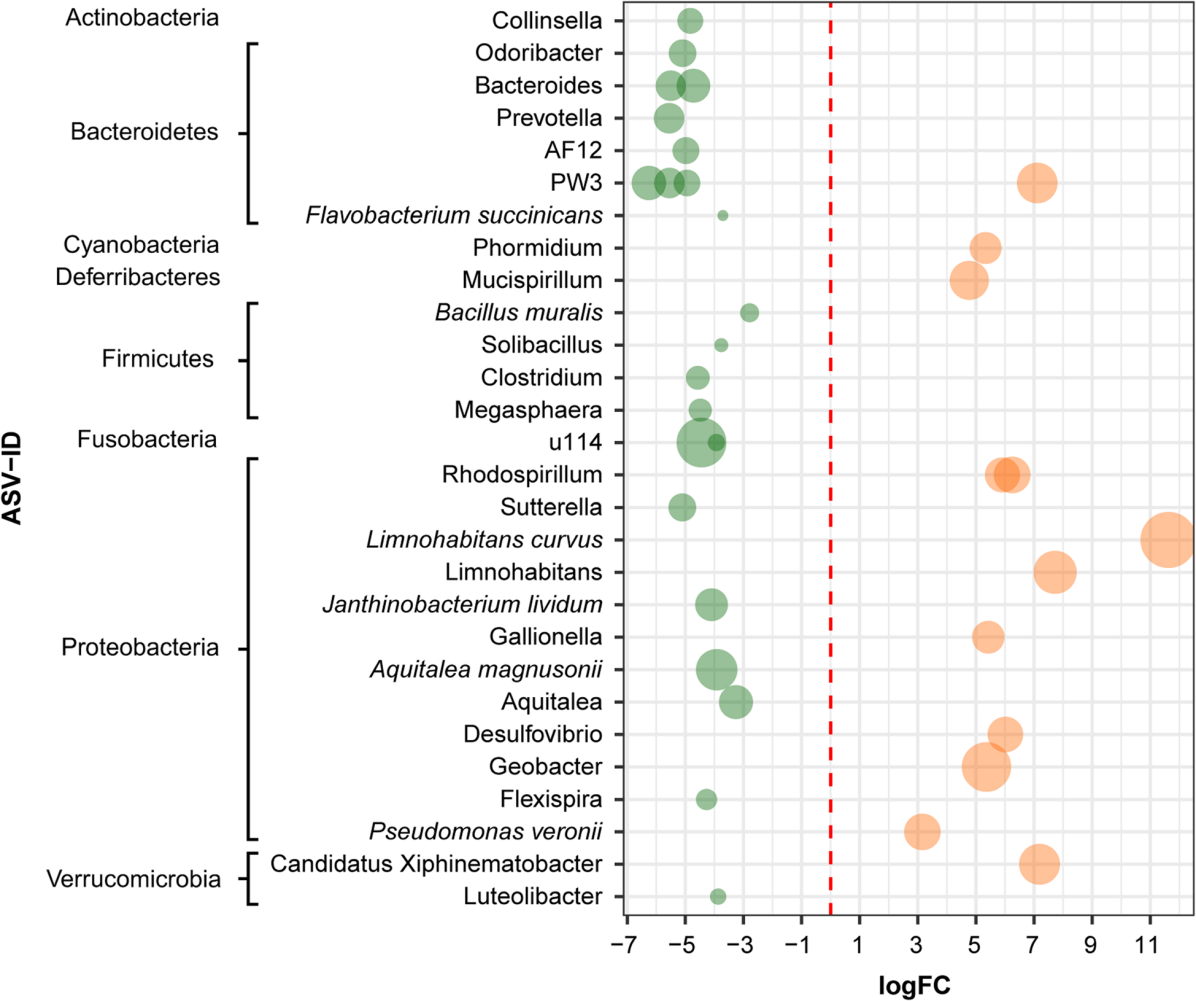
Fold-changes of bacterial ASVs detected in tadpoles that differ between undisturbed/disturbed habitats. Bacterial ASV with a log2 fold change less than 0 were more abundant in the undisturbed habitat indicated on the left and colored on green, whereas those with a log2 fold change higher than 0 were more abundant in disturbed habitat indicated on the right and colored on orange. Bacterial ASVs (circles) are sized by mean relative abundance across samples. Only the ASVs identified at genus and species level are shown. See Additional file 3: Table S5 for complete results of edgeR analysis and detailed taxonomic affiliation

EdgeR analysis detected 75 and 106 ASVs in the skin of tadpoles and adults, respectively, which differed significantly in abundance between undisturbed and disturbed habitats (Additional file 3: Table S5 and Table S6). In tadpoles, we observed an increase in abundance of 39 ASVs in the undisturbed habitats and 36 ASVs in the disturbed habitats. Some ASVs with higher abundance in tadpoles in the undisturbed habitats belonged to the order Burkholderiales, the families Planococcaceae and Victivallaceae, and to genera PW3 and Petrovella (Fig. 3, Additional file 3: Table S5). On the contrary, the ASVs that show a significantly higher abundance in tadpoles in the disturbed habitats belong to several families such as Comamonadaceae and Ruminococcaceae, and to the genera Limnohabitans, Geobacter and Rhodospirillum (Fig. 3, Additional file 3: Table S5).

**Fig. 4:**
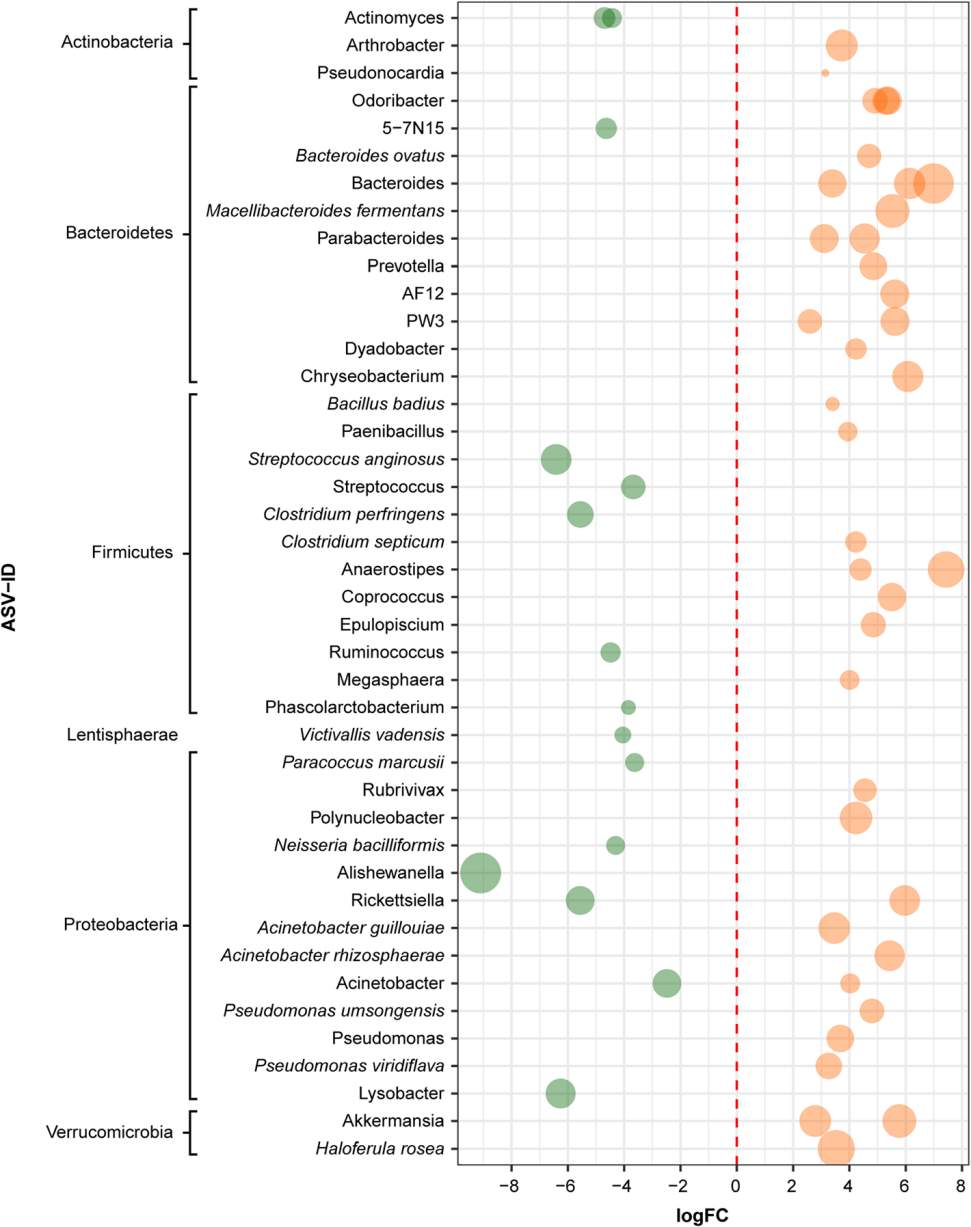
Fold-changes of bacterial ASVs detected in adults that differ between undisturbed/disturbed habitats. Bacterial ASV with a log2 fold change less than 0 were more abundant in the undisturbed habitats indicated on the left and colored on green, whereas those with a log2 fold change higher than 0 were more abundant in disturbed habitats indicated on the right and colored on orange. Bacterial ASVs (circles) are sized by mean relative abundance across samples. Only the ASVs identified at genus and species level are shown. See Additional file 3: Table S6 for complete results of edgeR analysis and detailed taxonomic affiliation

In adults, 39 ASVs were more abundant in undisturbed habitats and 67 ASVs in disturbed habitats. Some of the ASVs with significantly higher abundance in adults in the undisturbed habitats belonged to the families Ruminococcaceae and Nostocaceae, and to the genera Streptococcus and Actinomyces (Fig. 4, Additional file 3: Table S6). On the other hand, the ASVs showing significantly higher abundance in the disturbed habitats belonged to the families Lachnospiraceae, Bacteroidaceae and Comamonadaceae, and to the genera Bacteroides, Acinetobacter, Odoribacter and Pseudomonas (Fig. 4, Additional file 3: Table S6).

### Putative *Bd*-inhibitory bacterial community of adults of *Lithobates vibicarius* between undisturbed and disturbed habitats

**Fig. 5:**
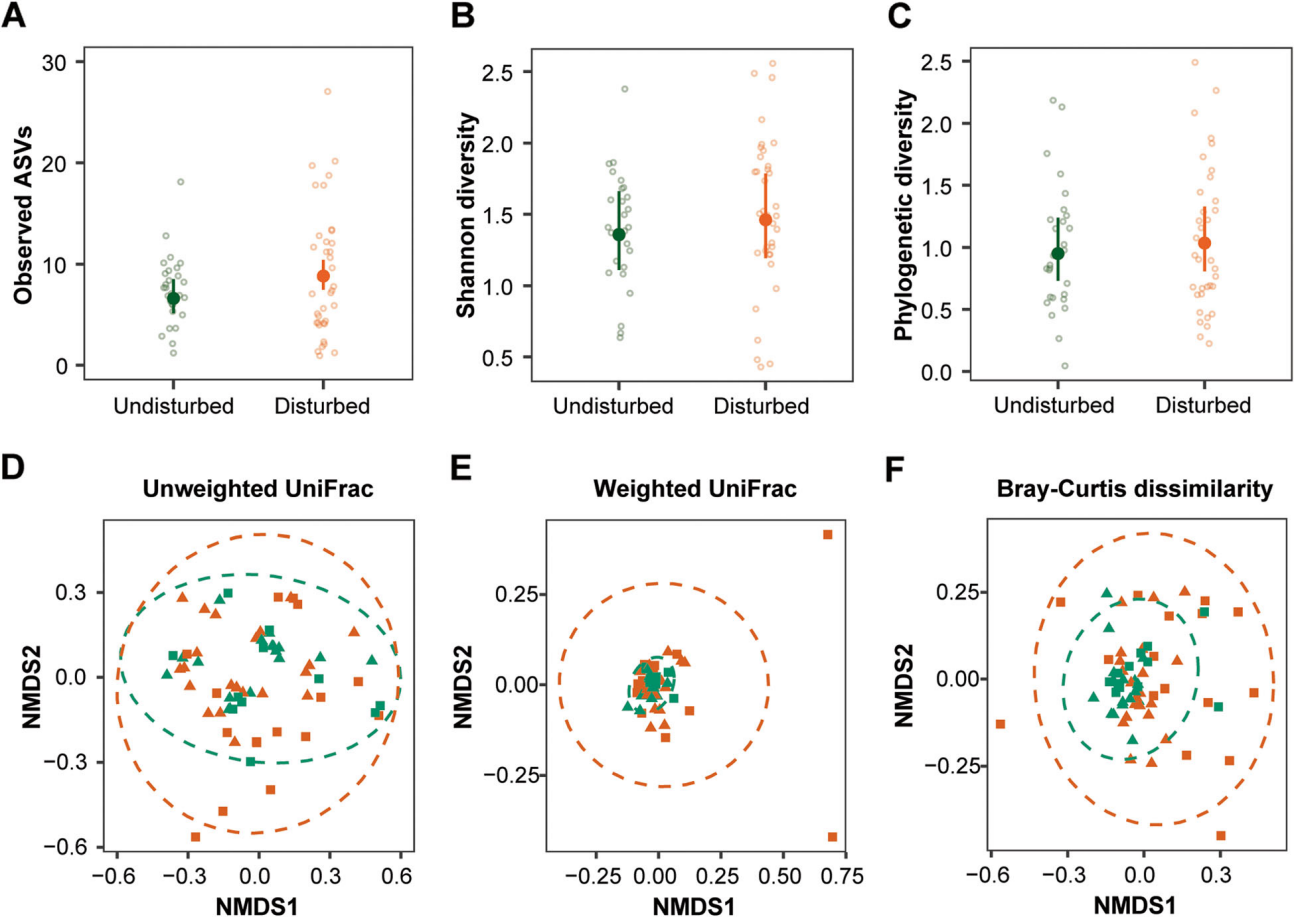
Diversity of the putative *Bd*-inhibitory bacterial community of *L. vibicarius* between undisturbed/disturbed habitats. Putative *Bd*-inhibitory bacterial alpha diversity of adults between habitat types (**a**-**c**). Lines indicate 95% confidence intervals (95% CIs). The Non-Metric Multidimensional Scaling plots (NMDS) of putative *Bd*-inhibitory bacterial beta diversity of adults regarding habitat types (**d**-**f**). Each light point represents the *Bd*-inhibitory bacterial ASV community of an individual; point color indicates habitat types (green - undisturbed habitats and orange - disturbed habitats) and shape indicates year of survey (square - year 2016 and triangle – year 2017). Ellipses show 95% CIs for each habitat type

The putative *Bd*-inhibitory bacterial community followed a similar pattern in alpha diversity as the entire bacterial community across habitat types, except for the number of observed ASVs with potential *Bd* inhibitory function, which showed a difference between undisturbed and disturbed habitats (Fig. 5a-c, Additional file 4: Table S7). Specifically, adults from the disturbed habitats showed a higher number of observed ASVs with potential *Bd* inhibitory function than those from undisturbed habitats (OR = 1.33, 95% CI = 1.02–1.75, Fig. 5a). The year of survey explained part of the variation for the observed ASVs and the Shannon diversity (Additional file 4: Table S7). Regarding beta diversity, the putative *Bd*-inhibitory bacterial community between habitat types differed when unweighted UniFrac (R^2^ = 0.02, *p* = 0.001) and Bray-Curtis dissimilarity (R^2^ = 0.06, *p* = 0.001) were used, while it remained similar when weighted UniFrac was used (R^2^ = 0.04, *p* = 0.09) (Fig. 5d-f, Additional file 4: Table S8). The year of survey explained part of the variation for the unweighted UniFrac and Bray-Curtis dissimilarity (Additional file 4: Table S8). The NMDS plot showed that adults in sites without habitat disturbance clustered more closely than those in sites with habitat disturbance (Additional file 4: Fig. S4).

**Fig. 6:**
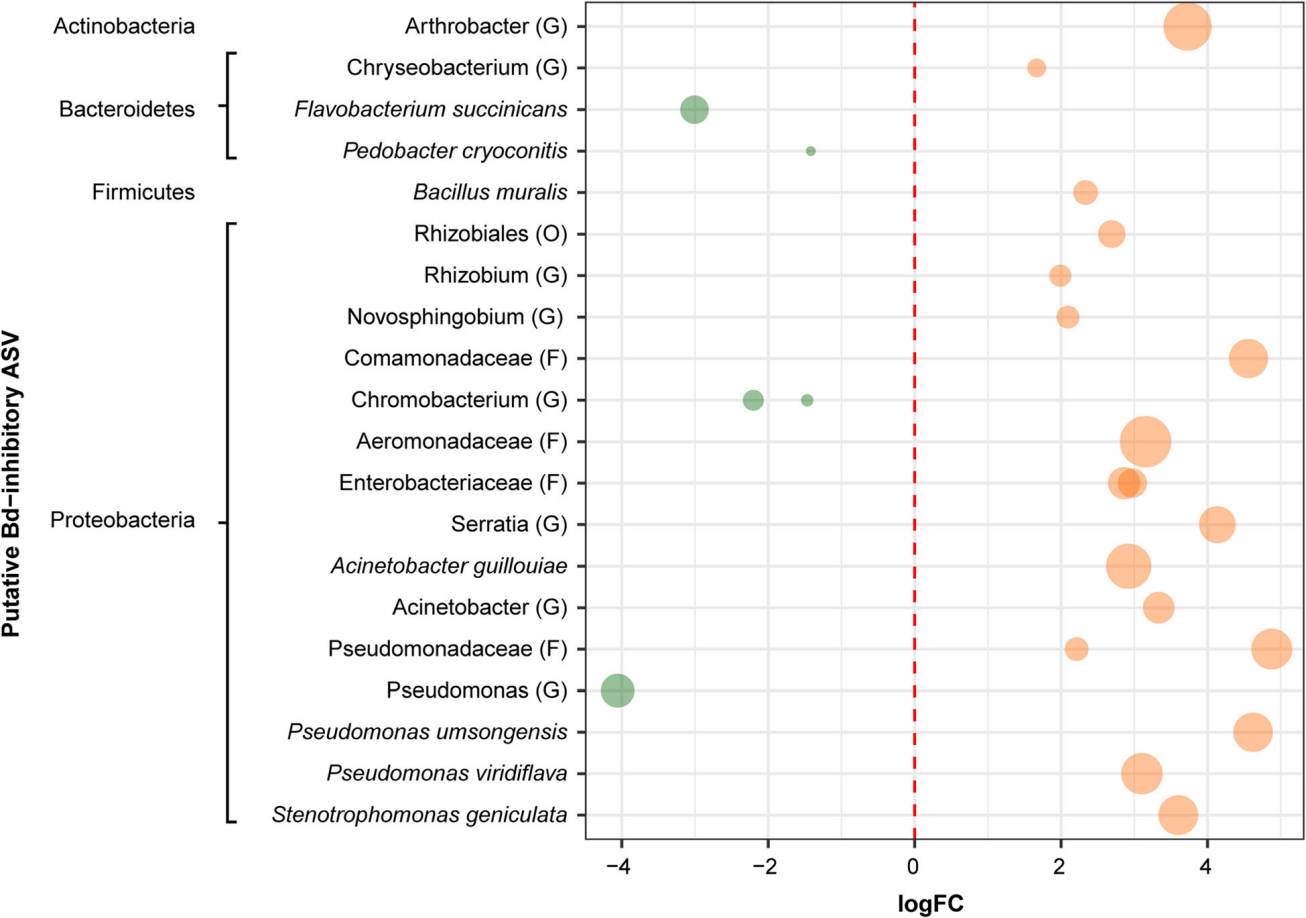
Fold-changes of putative *Bd*-inhibitory bacterial ASVs detected in adults that differ between undisturbed/disturbed habitats. Bacterial ASV with a log2 fold change less than 0 were more abundant in the undisturbed habitats indicated on the left and colored on green, whereas those with a log2 fold change higher than 0 were more abundant in disturbed habitats indicated on the right and colored on orange. Bacterial ASVs are sized by mean relative abundance across samples. The highest possible taxonomic assignment (maximal to the species level) is shown for each ASV. G, Genus; F, Family; O, Order

Noteworthy, the edgeR analysis revealed important differences in the abundance pattern of putative *Bd*-inhibitory ASVs between adults in undisturbed and disturbed habitats (Fig. 6). We detected 18 putative *Bd*-inhibitory ASVs with significantly higher abundance in adults in disturbed habitats, whereas only five showed this difference in adults in undisturbed habitats (Fig. 6). Part of these ASVs of adults in the disturbed habitats belong to the genera Acinetobacter, Pseudomonas and Arthrobacter*,* and to the species *Acinetobacter guillouiae*, *Pseudomonas umsongensis*, *P. viridiflava*.

## Discussion

Using high-throughput amplicon sequencing, we provide evidence that habitat disturbance, which involves cattle grazing lands, agriculture activities, and loss of natural forest coverage can lead to changes in the natural range of variation of the amphibian skin microbiome, in an endangered species with suspected evolution of *Bd* resistance. Due to the involvement of the microbiome in host health, understanding how anthropogenic disturbance modifies host microbiomes and the extent to which this can impact the host health, provide us with relevant information to create healthier environments for amphibians [35].

We hypothesized that habitat disturbance would influence the skin microbiome of *L. vibicarius*. Our results show that the alpha and beta diversity of the tadpole and adult skin bacterial communities differed between undisturbed and disturbed habitats. In addition, the relative abundance of certain bacterial taxa on the skin of both life stages differed between the two habitat types. These microbiome changes are congruent with other studies with different taxa assessing the effects of environmental changes (e.g., the gut microbiota of Darwin’s finches in the Galapagos islands [42]) [11, 18, 42]. It is known that human activities have an impact on the abiotic conditions of the environment affecting the diversity of host bacterial communities. For example, water and soil characteristics have shown to influence the amphibian skin bacterial communities across distinct habitat types [37, 43]. Further, environmental conditions cause changes to the environmental microorganisms (e.g., soil and water bacteria) [22], including those that can colonize hosts. Therefore, the environmental conditions in our undisturbed and disturbed habitats may be directly and/or indirectly driving the diversity in the skin microbiome of *L. vibicarius*. Human activities can also increase host stress, affecting corticosterone levels that can induce changes in the composition of the microbial communities [23]. These patterns could be responsible for generating the observed differences in *L. vibicarius* skin microbiomes between undisturbed and disturbed habitats. Our results support the idea that habitat disturbance influences the skin microbiome structure of an endangered neotropical-montane frog, but due to low sample size (e.g., number of study sites), particularly for tadpoles, and the correlative nature of our data, our results need further elaboration. The role of abiotic factors and environmental bacterial symbionts in the amphibian skin microbiota variation are relevant for consideration in future work towards a better understanding of environmental changes in the skin microbiome variation of *L. vibicarius*.

We found that the number of observed ASVs and Shannon diversity in individuals of tadpoles from the disturbed habitats was lower than in those from the undisturbed habitats. Further, our data revealed significant differences of the beta diversity of the skin microbial communities in tadpoles and adults between habitat types. The low number of bacterial strains and reduced diversity of the microbiome may contribute to an increased susceptibility to opportunistic pathogens and poor health [41, 44, 45]. According to Bletz et al. [46], differences in the overall diversity of the microbial community also reflected differences in functional performance that may reflect variation in both presence and strength of host-microbe interactions (e.g., production of antimicrobial metabolites to defend the host from pathogens). If this is the case with *L. vibicarius* in this study, further detriment to habitat quality and the introduction of novel microbiota by livestock (e.g. potential pathogens) could cause health detriment to the host.

We observed that individuals in the disturbed habitats, particularly adult frogs, showed a bacterial community with higher dispersion than those found in the undisturbed habitats. This pattern led us to explore the “Anna Karenina Principle” (AKP) for disturbed microbiomes [9], which aims at a better understanding of host resilience, as the conformation of the microbiome can influence hosts fitness and health. The pattern of increased dispersal of *L. vibicarius* in disturbed habitats is suggestive of the effects of microbial AKP. These are characterized by a greater variation in individual profiles that may be to an increased hosts stress due to the presence of environmental stressors in the disturbed habitats that alters a stable state of the microbiome in unpredictable ways that affect host health [7, 9]. Previous studies have also shown AKP effects on microbiomes of wildlife species (e.g., the gut microbiome of macaques in contact with humans [47]). We cannot definitively determine whether AKP effects occur without longitudinal sampling, however, our initial findings suggest these effects. Since the skin microbiota of tadpoles and adults in disturbed habitats appear to be in a dysbiotic state relative to those in undisturbed habitats, they could have an increased susceptibility to pathogenic infections that threaten the health of the host [41, 48]. This is particularly important because there is increasing evidence that skin and non-skin pathogens affect the skin microbiota of amphibians [45, 47, 49], and amphibians in pastures are infested by a greater richness and abundance of parasites [50]. Although these explanations are intriguing and very plausible, a semi-controlled field experiment would be necessary to corroborate this hypothesis.

It is known that pathogens can cause both exacerbated microbial variability/dispersion and increased susceptibility to pathogenic infections (e.g., *Salmonella typhimurium* in the mouse [51] and *Bd* in the bullfrog [52]. Infection by other pathogens beyond *Bd* (e.g., *Aeromonas hydrophila*, *Saprolegnia* spp.), and even co-infections (e.g., *Bd* and Ranaviruses [53]) may increase the level of cutaneous microbial dispersion in *L. vibicarius* in disturbed habitats, causing unpredictable host health impacts. Further research with multiple surveys across the different reproductive periods of *L. vibicarius* will be essential to study these interactions, which will increase considerable our understanding of disease dynamics under a new approach, the disease pyramid (host – pathogen – microbiome – environment [54]).

According to our predictions, we observed different patterns in the diversity of the putative *Bd*-inhibitory bacterial community of adults between the two habitat types. The adults in disturbed habitats showed a greater richness of putative *Bd*-inhibitory bacteria than those in undisturbed habitats. Also, adults in disturbed habitats had a higher number of putative *Bd*-inhibitory bacteria with higher relative abundance than those in undisturbed habitats. We cannot conclude that these patterns of the bacterial community are part of the defense mechanism against *Bd*. However, a previous study found a higher proportion of pathogen-resistant bacteria in frogs inhabiting farms suggesting a higher occurrence rate of harmful pathogens in the farmland habitat [16]. Therefore, it is also possible that *L. vibicarius* remains at risk from *Bd* in the disturbed habitats. Besides that, previous studies indicate that richer and more diverse communities show greater functionality in protecting the host against *Bd* [40, 41, 55]. Furthermore, it is known that the relative abundance patterns of different protective bacterial taxa influence the protective function against invading pathogens, and the bacterial diversity patterns can determine in part the production of some metabolites against *Bd* by these symbionts [32, 56]. Based on this evidence, we suggest that both the high richness and the increased number of *Bd*-inhibitory bacterial strains with high relative abundance can be enhancing protection against *Bd* in adults of *L. vibicarius* in disturbed habitats. We should also consider the possibility of a functional redundancy of these *Bd*-inhibitory bacterial communities between adults in the two habitat types. A previous study found that the composition of the *Bd*-inhibitory bacterial community, using only bacterial isolates, of the salamander *Plethodon cinereus* changed along an anthropogenic land-use gradient [57]. The findings of Barnes et al. [57] indicate that this type of pattern does not correspond to a change in *Bd*-inhibitory function, on the contrary it is suggestive of a functionally redundant characteristic along a gradient of habitat change. Our results highlight the need of future research to investigate the anti-*Bd* functionality of the microbiome of *L. vibicarius* using a transcriptomic approach.

The presence of adult individuals in the disturbed habitats with the highest richness of putative *Bd*-inhibitory bacteria may help to understand why anthropogenic habitat loss is negatively associated with *Bd* in amphibian populations, along with the hypothesis that suboptimal microclimate conditions for *Bd* in disturbed habitats is a potential mechanism for reducing *Bd* infections, as suggested by Becker and Zamudio [58]. However, this should be interpreted with caution, because this effect could only be applied within species that can so far tolerate anthropogenic activities, and the level of disturbance in our study sites may not have seriously affected the functionality of the microbiome to protect against *Bd*. If the level of anthropogenic environmental stressors increases and the environmental conditions of the habitat change dramatically, it could be that the functionality of the *Bd*-protective bacterial community is disrupted, along with the proper functioning of the host immune system. This could therefore cause the spread of hosts that develop chytridiomycosis and increase the risk of extinction.

Besides the influence of habitat disturbance on the skin bacterial community of tadpoles and adults of *L. vibicarius*, we found temporal variation in these symbionts (i.e., between years of survey). The effect of time playing a role shaping the microbial communities is consistent with other studies showing a variation in the bacterial communities across short and long time scales [59, 60]. The observed community variation over time is likely associated with fluctuations in external environmental conditions, such as rainfall, humidity and temperature [60, 61]. Here, we also observed temporal variation in the putative *Bd*-inhibitory community that could suggest a disruption of the protective function, but also that functional redundancy could be occurring. Extrinsic factors from the environment are known to determine the stability of the host microbiome over time, which may therefore have relevant consequences for the stability of host-microbiome relationships and microbiome-fitness correlations [45]. More research is needed to elucidate the relevance of temporal variation in the skin bacterial communities to better understand factors influencing host-associated skin bacterial communities in the context of habitat disturbance and climate change. For example, an increment of livestock grazing across time can increase the amount of cattle feces, which can enhance the environment for novel pathogenic microorganisms that amphibians can acquire into their microbial community. Due to potential increment of anthropogenic pressures in the disturbed habitats of our study sites across years, a long-term microbiome surveillance will be relevant for exploring the dynamics (year-to-year variation) of putative *Bd*-inhibitory ASVs, in terms of survival and proliferation, especially for amphibian conservation efforts.

## Conclusions

Our study suggests that habitat alteration influences the skin microbiome of *L. vibicarius*. Our findings are in line with the Anna Karenina Principle, which is an important response of animal microbiomes to stress factors that decrease the ability of the host or its microbiome to regulate community composition. The observed pattern of microbiome dispersion may be due to the presence of environmental stressors apart from pathogens, which can perturb a stable state leaving animals more susceptible to pathogen infections even beyond *Bd*. Our results further suggest that despite signs of a dysbiotic state in the disturbed habitats, the species inhabiting these habitats seems to harbor a *Bd*-inhibitory community with characteristics that enhance protection against *Bd*. The latter could be explained by the development of a defense mechanism to improve *Bd*-protection, attributed to the co-occurrence of host and pathogen for more than 30 years in these habitats [62]. However, we suspect that if the level of anthropogenic stressors increases, the *Bd*-protective bacterial community could be altered along with its functionality, putting the health of the host at an increased risk again. Despite limitations of the study due to low sample sizes, our results emphasize the importance of careful managements of landscapes outside protected areas to ensure the health of the endangered amphibian populations, particularly because of the potential introduction of new *Bd* bacterial strains and novel pathogens. Finally, our study contributes to efforts to develop microbial indices and baselines that can be used to manage or manipulate the amphibian skin microbiome to better protect host health across localities with different human use.

## Methods

### Study sites and sampling

The study was conducted at six sites in the high-altitude cloud forest of Juan Castro Blanco National Park and adjacent private lands within the province of Alajuela, Costa Rica (10° 16′ 44.4“ N, 84° 19’ 58.9” W). The elevation of the study sites ranges from 1850 to 2100 m.a.s.l. We have not provided the exact coordinates of the study sites to discourage people from visiting them, because we hope to prevent the possible introduction of pathogens and illegal collecting.

The study sites were monitored for the presence of *L. vibicarius* for two consecutive years (2016–2017) during the rainy season (September–November). Three study sites (Lagunillas, Monjes and Congo) represent areas in disturbed landscapes adjacent to the National Park. Each of these sites contains a pond and small streams within the pastures; the pastures are managed habitats for livestock grazing and agricultural activities and are surrounded by secondary and fragmented mountain rainforest. In addition, the loss of natural forest cover, the presence of cow feces, antibiotic treatments to cows and the use of pesticides (e.g. herbicides) on these sites could adversely affect the quality of habitat for amphibians. The other three study sites (Pozo verde, Pozo seco and Tamara) are in the protected and largely undisturbed National Park. Each of these sites comprises a large pond surrounded by well-preserved late successional montane forests.

We captured tadpoles and adults of *L. vibicarius* and collected two skin swabs to analyze the skin bacterial community and the *Bd*-infection status, using previously published procedures [35]. We released the animals at the site of captured after swabbing. The swabs for bacterial analysis were stored in sterile 1.5 ml Eppendorf tubes with 300 μl of DNA/RNA Shield (Zymo Research). The swabs for *Bd* analysis were placed directly in dry 1.5 ml sterile Eppendorf tubes. All swabs were stored at − 20 °C until further processing. We obtained unequal sample sizes between the study sites for each life stage due to unfavorable conditions during fieldwork. Details on sample sizes per study site are summarized in Additional file 1: Table S1. We used only the samples that were negative for *Bd*. The presence of *Bd* DNA from swabs was assessed according to previously published procedures [53]. In a previous study, a subsample of the individuals was used to understand the variation of life history in the skin microbiome [35], whereas the focus of the present study was to investigate the influence of habitat disturbance on the skin microbiome.

### Molecular methods and sequencing

The bacterial genomic DNA from swabs was extracted using the NucleoSpin Soil (MACHEREY-NAGEL) following the manufacturer’s instructions and 16S rRNA gene amplicon sequencing methods were performed as described previously [35]. Briefly, we amplified the hypervariable V4 region of the 16S rRNA gene (291 bp) with the primer pair 515 F (5-GTGCCAGCMGCCGCGGTAA-3) and 806 R (5-GGACTACHVGGGTWTCTAAT-3). After purification (NucleoMag NGS Beads, Macherey-Nagel) and quantification (QuantiT™ PicoGreen® kit, Invitrogen-Life Technologies) of barcoded samples, the pooled sample library was sequenced as paired-end run on Illumina MiSeq platform at the Institute of Evolutionary Ecology and Conservation Genomics, Ulm University.

### Sequence data processing

The initial processing of the sequence reads was done using the open source software QIIME2 (version 2019.1) according to previously published procedures [35]. The sequences were assigned to amplicon sequence variants (ASVs) and assigned to the taxonomy using the Greengenes database (version 13_8) as reference (http://greengenes.lbl.gov). Any sequence classified as chloroplast, mitochondria, archaea, eukaryota and unclassified phylum was excluded. We imported our data into R environment (version 3.4.4) [63] for further processing of the sequences using the R package “phyloseq” [64]. Samples with less than 8000 sequences were excluded. We also eliminated any ASV with less than 10 sequences. For alpha and beta diversity analyses, we normalized the read counts between samples by rarefying the datasets according to the sample with the lowest number of reads among samples.

### Statistical analysis

To assess alpha diversity, we calculated three different metrics for each sample using the R packages “phyloseq” and “picante”: skin microbial community richness (i.e., the number of observed ASVs), ASVs diversity (i.e., the Shannon diversity), and phylogenetic diversity (i.e., Faiths phylogenetic diversity). We first performed Generalized Linear Models (GLMs) with a Gaussian distribution to test whether the alpha diversity of the tadpole skin bacterial communities differs between undisturbed and disturbed habitats. We incorporated the year of survey as a covariate in the GLMs to account for annual variation. Then, to assess whether the alpha diversity of the adults differs between undisturbed and disturbed habitats, we performed Generalized Linear Mixed Models (GLMMs) with a Gaussian error structure. Again, we incorporated the year of the study as a covariate and implemented study site identity as a random factor to control for non-independence of samples from the same site. Alpha diversity measures were log-transformed prior to analysis to improve normality. We determined the significance of covariates using the *Anova* function with χ^2^ as the test statistic. We used the odds ratio (OR) and 95% confidence intervals (95% CI) [43] to quantify the deviation (i.e., the effect size) of the alpha diversity metrics across habitats. Odds ratios and 95% CI were estimated using the R package “emmeans” [65].

We calculated three separate beta diversity metrics: unweighted UniFrac (phylogenetic presence/absence-based differences), weighted UniFrac (phylogenetic abundance-based differences) and Bray-Curtis dissimilarity (non-phylogenetic abundance-based differences) to investigate the influence of habitat disturbance on beta diversity of tadpole and adult skin microbiomes. We excluded ASVs with 10% prevalence or less, as beta diversity analyses are sensitive to rare ASVs variation. These measurements were calculated within the R package “phyloseq”. We fitted Permutational Multivariate Analysis of Variance (PERMANOVA) using the *adonis* function of the R package “vegan” [66] to test whether the measure of beta diversity differs significantly between habitat types. In the tadpole and adult analyses, we incorporated the year of survey as a covariate to account its variation. In the adult analyses, we included study site identity in the *strata* argument of the *adonis* function to consider the nesting effect. Due to the lack of tadpole samples at some sites during the two years of study, we could not include study site identity in the *strata* argument for the PERMANOVA analyses with tadpole samples. However, we performed a PERMANOVA using the Bray-Curtis dissimilarity and included study sites and year of study as covariates. Moreover, we used Non-Metric Multidimensional Scaling (NMDS) plots to visualize beta-diversity patterns using the R package “phyloseq”.

We performed multivariate of homogeneity of group dispersion analysis to evaluate how habitat types influences dispersal in bacterial communities among individuals in the same group (inter-individual distances) using the three beta diversity metrics. For each metric, we used a GLM with a Gaussian error structure to test the differences in the point-to-center distances between the microbial communities on the skin of individuals from undisturbed and disturbed habitats. We used the ORs to estimate the effect size between the habitats as described above.

Empirical Analysis of Digital Gene Expression Data (hereafter edgeR analysis) within the R package “*edgeR*” [67] was used to identify bacterial ASVs in both tadpoles and adults that significantly differed in abundance between undisturbed and disturbed habitat (FDR-corrected *p*-values at *p* < 0.001). For these analyses, we used the unrarefied dataset and the method Trimmed Mean of M-values (TMM) for normalization of samples.

We examined the community of putative *Bd*-inhibitory bacterial ASVs among habitat types as we did previously for the entire adult skin bacterial community of adults. For alpha and beta diversity, we followed the same statistical framework as above. To explore the variation of these bacterial strains between undisturbed and disturbed habitats, we performed an edgeR analysis to identify ASVs with differential abundance between habitat types. To identify putative *Bd*-inhibitory bacterial ASVs, we filtered the ASVs tables to retain only those reads that matched 100% sequence identity to those from the database of cultivable anti-*Bd* bacteria “Antifungal Isolates Database” [68]. Note that this does not necessarily mean that these bacterial strains possess anti-*Bd* properties, but that they might be candidates [69].

## Supplementary information

**Additional file 1 : Table S1**. Samples of *Lithobates vibicarius* used for microbiome analysis. Samples with > 8000 reads.

**Additional file 2 : Table S2.** Summary of GLMs and GLMMs predicting alpha diversity metrics in tadpoles and adults, respectively. Habitat types and year of survey were use as covariates. Study site was used as a random factor in the GLMMs. Significant *p* values (< 0.05) are shown in bold. **Fig. S1.** Alpha diversity metrics among tadpoles (A-C) and adults (D-F) regarding habitat types. Each light point represents the bacterial skin community of an individual sample; point color indicates habitat type (green - undisturbed habitat and orange - disturbed habitat). Lines indicate 95% confidence intervals (95% CIs). **Table S3.** Summary of PERMANOVAs of beta diversity metrics in tadpoles and adults. Habitat types and year of survey were use as covariates and study site were used as covariates. Study site was used as a random factor in the analysis of adults. Significant *p* values (< 0.05) are shown in bold. **Table S4.** Summary of PERMANOVAs of beta diversity in tadpoles. Study types and year of survey were use as covariates. Significant *p* values (< 0.05) are shown in bold. **Fig. S2.** Beta diversity of *L. vibicarius* skin microbiota among study sites and year of survey. The Non-Metric Multidimensional Scaling plots (NMDS) of the beta diversity of the microbiota of tadpoles (A) and adults (B) were based on Bray-Curtis dissimilarity. Each point represents the bacterial community of an individual; point color indicates study sites and shape indicates year of survey.

**Additional file 3 : Table S5.** Fold-changes of bacterial ASVs detected on the skin of tadpoles that differ between undisturbed/disturbed habitats. **Table S6.** Fold-changes of bacterial ASVs detected on the skin of adults that differ between undisturbed/disturbed habitats.

**Additional file 4 : Table S7.** Summary of the GLMMs predicting putative *Bd*-inhibitory bacterial alpha diversity metrics in adults. Habitat types and year of survey were use as covariates and study site was used as a random factor in the models. Significant *p* values (< 0.05) are shown in bold. **Table S8.** Summary of PERMANOVAs of the putative *Bd*-inhibitory bacterial beta diversity in adults. Habitat types and year of survey were use as covariates and study site was used as a random factor in the models. Significant *p* values (< 0.05) are shown in bold. **Fig. S4.** Beta diversity of putative *Bd*-inhibitory bacterial community on adults across sites and year of survey. The Non-Metric Multidimensional Scaling plots (NMDS) of the beta diversity of the microbiota of adults were based on Bray-Curtis dissimilarity. Each point represents the bacterial community of an individual; point color indicates study sites and shape indicates year of survey.

## Data Availability

The 16S rRNA sequences used in this study are available in the Dryad Digital Repository 10.5061/dryad.n34035p

## Abbreviations

ASV: Amplicon sequence variant
*Bd*: *Batrachochytrium dendrobatidis*
AKP: Anna Karenina principle
GLM: Generalized Linear Models
GLMM: Generalized Linear Mixed Models
PERMANOVA: Permutational Multivariate Analysis of Variance
TMM: Trimmed Mean of M-values
NMDS: Non-Metric Multidimensional Scaling
OR: odds ratio
