# Supplementary material for: Habitat disturbance influences the skin microbiome of a rediscovered neotropical-montane frog
Source: BMC Microbiol. 2020 Sep 22;20:292. doi: 10.1186/s12866-020-01979-1 (PMC7509932; doi:10.1186/s12866-020-01979-1)
Supplement: Supplementary file 1 — Additional file 1 : Table S1. Samples of Lithobates vibicarius used for microbiome analysis. Samples with > 8000 reads. [file 12866_2020_1979_MOESM1_ESM.docx]

**Additional file 1: Supplementary Material**

**Table S1**. Samples of *Lithobates vibicarius* used for microbiome analysis. Samples with > 8000 reads.

| Datasets | Site | Year | #Samples |
| --- | --- | --- | --- |
| Tadpole dataset | Congo | 2016 | 6 |
|  | Lagunillas | 2017 | 7 |
|  | Monjes | 2017 | 11 |
|  | Pozo seco | 2016 | 3 |
|  |  | 2017 | 6 |
|  | Tamara | 2016 | 3 |
|  |  | 2017 | 5 |
| Adult dataset | Congo | 2016 | 5 |
|  |  | 2017 | 9 |
|  | Lagunillas | 2016 | 6 |
|  |  | 2017 | 10 |
|  | Monjes | 2016 | 6 |
|  |  | 2017 | 3 |
|  | Pozo seco | 2016 | 3 |
|  |  | 2017 | 8 |
|  | Tamara | 2016 | 5 |
|  |  | 2017 | 4 |
|  | Pozo verde | 2016 | 4 |
|  |  | 2017 | 5 |
