# Supplementary material for: Habitat disturbance influences the skin microbiome of a rediscovered neotropical-montane frog
Source: BMC Microbiol. 2020 Sep 22;20:292. doi: 10.1186/s12866-020-01979-1 (PMC7509932; doi:10.1186/s12866-020-01979-1)
Supplement: Supplementary file 2 — Additional file 2 : Table S2. Summary of GLMs and GLMMs predicting alpha diversity metrics in tadpoles and adults, respectively. Habitat types and year of survey were use as covariates. Study site was used as a random factor in the GLMMs. Significant p values (< 0.05) are shown in bold. Fig. S1. Alpha diversity metrics among tadpoles (A-C) and adults (D-F) regarding habitat types. Each light point represents the bacterial skin community of an individual sample; point color indicates habitat type (green - undisturbed habitat and orange - disturbed habitat). Lines indicate 95% confidence intervals (95% CIs). Table S3. Summary of PERMANOVAs of beta diversity metrics in tadpoles and adults. Habitat types and year of survey were use as covariates and study site were used as covariates. Study site was used as a random factor in the analysis of adults. Significant p values (< 0.05) are shown in bold. Table S4. Summary of PERMANOVAs of beta diversity in tadpoles. Study types and year of survey were use as covariates. Significant p values (< 0.05) are shown in bold. Fig. S2. Beta diversity of L. vibicarius skin microbiota among study sites and year of survey. The Non-Metric Multidimensional Scaling plots (NMDS) of the beta diversity of the microbiota of tadpoles (A) and adults (B) were based on Bray-Curtis dissimilarity. Each point represents the bacterial community of an individual; point color indicates study sites and shape indicates year of survey. [file 12866_2020_1979_MOESM2_ESM.docx]

**Additional file 2: Supplementary Material**

**Table S2.** Summary of GLMs and GLMMs predicting alpha diversity metrics in tadpoles and adults, respectively. Habitat types and year of survey were use as covariates. Study site was used as a random factor in the GLMMs. Significant p values (<0.05) are shown in bold.

| **Life stage** | **Variables** | **LR Chisq** | **DF** | ***p*-value** |
| --- | --- | --- | --- | --- |
| Tadpoles | **Number of observed ASVs** |  |  |  |
|  | Habitat type | 6.549 | 1 | **0.010** |
|  | Year of survey | 29.027 | 1 | **< 0.001** |
|  | **Shannon diversity** |  |  |  |
|  | Habitat type | 6.837 | 1 | **0.008** |
|  | Year of survey | 2.060 | 1 | 0.151 |
|  | **Faith's phylogenetic diversity** |  |  |  |
|  | Habitat type | 1.085 | 1 | 0.297 |
|  | Year of survey | 23.459 | 1 | **< 0.001** |
| Adults | **Number of observed ASVs** |  |  |  |
|  | Habitat type | 0.069 | 1 | 0.793 |
|  | Year of survey | 0.124 | 1 | 0.730 |
|  | **Shannon diversity** |  |  |  |
|  | Habitat type | 1.610 | 1 | 0.205 |
|  | Year of survey | 0.002 | 1 | 0.970 |
|  | **Faith's phylogenetic diversity** |  |  |  |
|  | Habitat type | 0.516 | 1 | 0.472 |
|  | Year of survey | 0.004 | 1 | 0.950 |

**Figure S1**


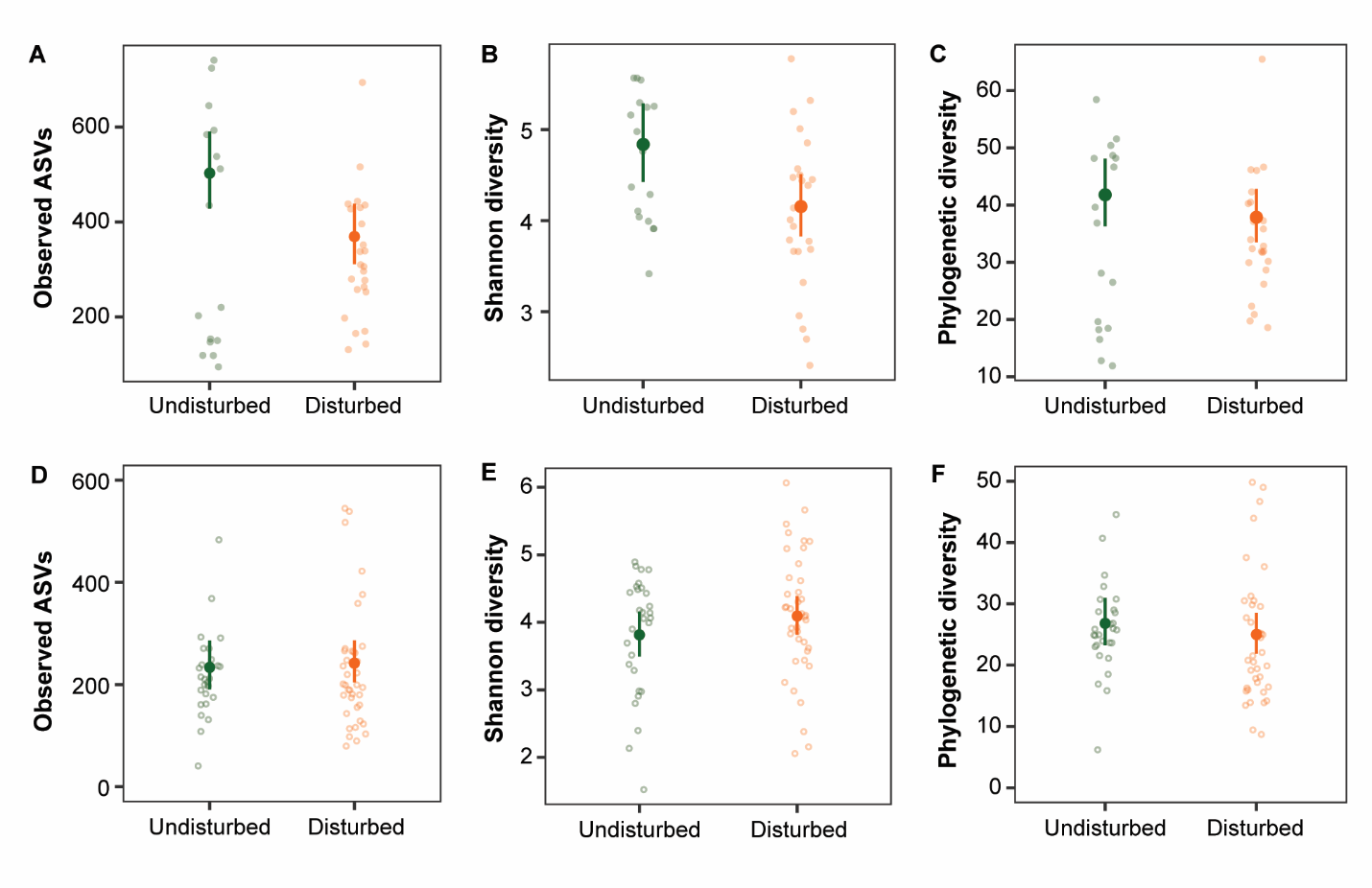


**Fig. S1.** Alpha diversity metrics among tadpoles (A-C) and adults (D-F) regarding habitat types. Each light point represents the bacterial skin community of an individual sample; point color indicates habitat type (green - undisturbed habitat and orange - disturbed habitat). Lines indicate 95% confidence intervals (95% CIs).

**Table S3.** Summary of PERMANOVA models of beta diversity metrics in tadpoles and adults. Habitat types and year of survey were use as covariates and study site were used as covariates. Study site was used as a random factor in the analysis of adults. Significant p values (<0.05) are shown in bold.

| **Variables** | **Unweighted UniFrac** | **Weighted UniFrac** | **Bray-Curtis dissimilarity** |
| --- | --- | --- | --- |
| **Tadpoles** |  |  |  |
| Habitat type | *F* = 3.57, ***p* = 0.001**, R^2^ = 0.07 | *F* = 6.91, ***p* = 0.001**, R^2^ = 0.14 | *F* = 7.12, ***p* = 0.001**, R^2^ = 0.14 |
| Year of survey | *F* = 8.70, ***p* = 0.001**, R^2^ = 0.17 | *F* = 5.02, ***p* = 0.003**, R^2^ = 0.10 | *F* = 3.60, ***p* = 0.001**, R^2^ = 0.07 |
| **Adults** |  |  |  |
| Habitat type | *F* = 5.54, ***p* = 0.004**, R^2^ = 0.08 | *F* = 9.06, ***p* = 0.049**, R^2^ = 0.12 | *F* = 5.47, ***p* = 0.001**, R^2^ = 0.07 |
| Year of survey | *F* = 3.22, ***p* = 0.004**, R^2^ = 0.04 | *F* = 2.12, ***p* = 0.049**, R^2^ = 0.03 | *F* = 3.30, ***p* = 0.001**, R^2^ = 0.04 |

**Table S4.** Summary of PERMANOVA models of beta diversity in tadpoles. Study types and year of survey were use as covariates. Significant p values (<0.05) are shown in bold.

| **Variables** | **Bray-Curtis dissimilarity** |
| --- | --- |
| Study sites | *F* = 5.57, ***p* = 0.001**, R^2^ = 0.36 |
| Year of survey | *F* = 3.49, ***p* = 0.001**, R^2^ = 0.05 |

**Figure S2**

**
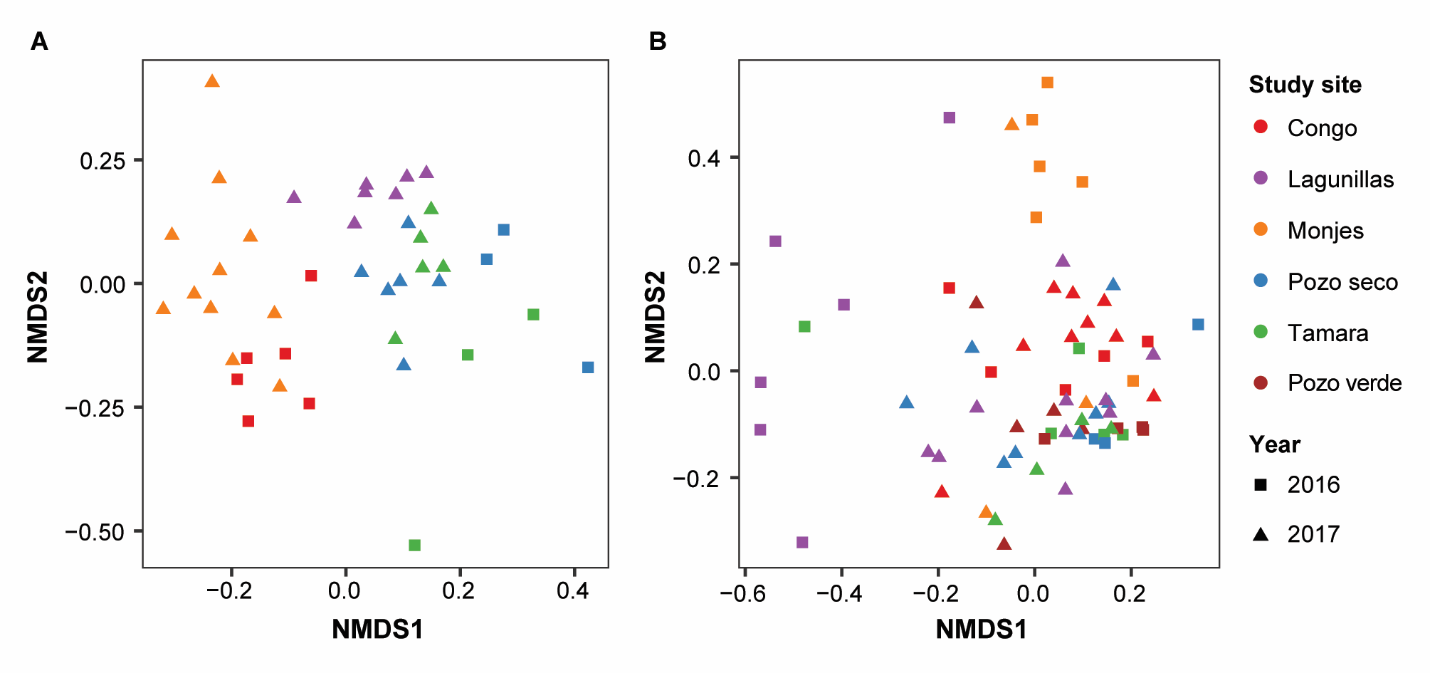
**

**Fig. S2.** Beta diversity of *L. vibicarius* skin microbiota among study sites and year of survey. The Non-Metric Multidimensional Scaling plots (NMDS) of the beta diversity of the microbiota of tadpoles (A) and adults (B) were based on Bray-Curtis dissimilarity. Each point represents the bacterial community of an individual; point color indicates study sites and shape indicates year of survey.
