# Supplementary material for: Habitat disturbance influences the skin microbiome of a rediscovered neotropical-montane frog
Source: BMC Microbiol. 2020 Sep 22;20:292. doi: 10.1186/s12866-020-01979-1 (PMC7509932; doi:10.1186/s12866-020-01979-1)
Supplement: Supplementary file 4 — Additional file 4 : Table S7. Summary of the GLMMs predicting putative Bd-inhibitory bacterial alpha diversity metrics in adults. Habitat types and year of survey were use as covariates and study site was used as a random factor in the models. Significant p values (< 0.05) are shown in bold. Table S8. Summary of PERMANOVAs of the putative Bd-inhibitory bacterial beta diversity in adults. Habitat types and year of survey were use as covariates and study site was used as a random factor in the models. Significant p values (< 0.05) are shown in bold. Fig. S4. Beta diversity of putative Bd-inhibitory bacterial community on adults across sites and year of survey. The Non-Metric Multidimensional Scaling plots (NMDS) of the beta diversity of the microbiota of adults were based on Bray-Curtis dissimilarity. Each point represents the bacterial community of an individual; point color indicates study sites and shape indicates year of survey. [file 12866_2020_1979_MOESM4_ESM.docx]

**Additional file 4: Supplementary materials**

**Table S7:** Summary of the GLMMs predicting putative *Bd*-inhibitory bacterial alpha diversity metrics in adults. Habitat types and year of survey were use as covariates and study site was used as a random factor in the models. Significant p values (<0.05) are shown in bold.

| **Variables** | **LR Chisq** | **DF** | ***p*-value** |
| --- | --- | --- | --- |
| **Number of observed ASVs** |  |  |  |
| Habitat types | 4.483 | 1 | **0.034** |
| Year of survey | 15.671 | 1 | **0.0001** |
| **Shannon diversity index** |  |  |  |
| Habitat types | 0.265 | 1 | 0.60 |
| Year of survey | 9.036 | 1 | **0.002** |
| **Faith's phylogenetic diversity** |  |  |  |
| Habitat types | 0.231 | 1 | 0.630 |
| Year of survey | 3.031 | 1 | 0.081 |

**Table S8.** Summary of PERMANOVA models of the putative *Bd*-inhibitory bacterial beta diversity in adults. Habitat types and year of survey were use as covariates and study site was used as a random factor in the models. Significant p values (<0.05) are shown in bold.

| **Variables** | **Unweighted UniFrac** | **Weighted UniFrac** | **Bray-Curtis dissimilarity** |
| --- | --- | --- | --- |
| Habitat types | *F* = 1.56, ***p* = 0.001**, R^2^ = 0.02 | *F* = 2.44, *p* = 0.09,  R^2^ = 0.04 | *F* = 4.37, ***p* = 0.001**, R^2^ = 0.06 |
| Year of survey | *F* = 3.83, ***p* = 0.001**, R^2^ = 0.05 | *F* = 1.65, *p* = 0.10,  R^2^ = 0.02 | *F* = 4.18, ***p* = 0.001**, R^2^ = 0.05 |

**Figure S4**

**
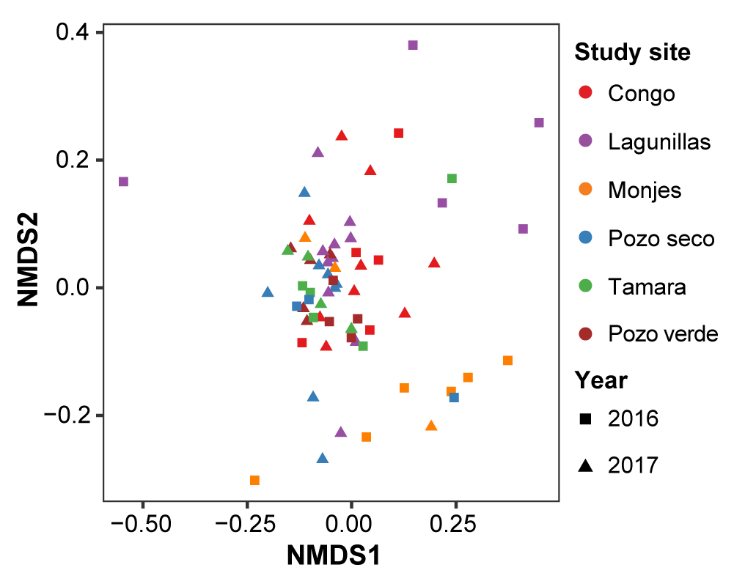
**

**Fig. S4.** Beta diversity of putative *Bd*-inhibitory bacterial community on adults across sites and year of survey. The Non-Metric Multidimensional Scaling plots (NMDS) of the beta diversity of the microbiota of adults were based on Bray-Curtis dissimilarity. Each point represents the bacterial community of an individual; point color indicates study sites and shape indicates year of survey.
